# Co‐pathology in Alzheimer's disease and Lewy body disease and its association with neuropsychiatric symptoms

**DOI:** 10.1002/alz.70693

**Published:** 2025-09-15

**Authors:** Lucy L. Gibson, Lea T. Grinberg, Victor R. Paes, Christoph Mueller, Renata E. P. Leite, Paula Villela Nunes, Alberto F. O. Justo, Carlos A. Pasqualucci, Eduardo Ferriolli, Dag Aarsland, Claudia K. Suemoto

**Affiliations:** ^1^ King's College London Centre of Healthy Brain Ageing Department of Psychological Medicine Institute of Psychiatry Psychology, and Neuroscience London UK; ^2^ Memory and Aging Center UCSF Weill Institute for Neurosciences University of California San Francisco California USA; ^3^ Department of Pathology University of California San Francisco California USA; ^4^ Department of Pathology University of São Paulo Medical School Av. Dr. Enéas de Carvalho Aguiar São Paulo Brazil; ^5^ Division of Geriatrics Department of Internal Medicine University of São Paulo Medical School Av. Dr. Enéas de Carvalho Aguiar São Paulo Brazil; ^6^ University of British Columbia 6200 University Blvd Vancouver British Columbia Canada; ^7^ Physiopathology in Aging Laboratory (LIM‐22) Department of Pathology University of São Paulo Medical School São Paulo Brazil; ^8^ Centre for Age‐Related Medicine Stavanger University Hospital Armauer Hansens vei 30 Stavanger Norway

**Keywords:** dementia with Lewy bodies, neuropathology, neuropsychiatric symptoms

## Abstract

**BACKGROUND:**

Mixed neuropathology is common in dementia, but the clinical implications for neuropsychiatric symptoms (NPSs) are not well characterized.

**METHODS:**

In a population‐based *post mortem* study, cases with Alzheimer's disease neuropathological change (ADNC) and Lewy body disease (LBD) were identified with any comorbid neuropathology (limbic‐predominant age‐related transactive response DNA‐binding protein 43 encephalopathy neuropathological change (LATE‐NC), cerebrovascular disease, LBD, and ADNC, respectively). *Post mortem* interviews collected information regarding NPSs and cognition to explore associations between each co‐pathology and NPSs across the whole cohort, as well as in participants without dementia.

**RESULTS:**

Co‐existing neuropathology was frequent, even among individuals without clinical dementia. In cases with ADNC, comorbid neocortical LBD pathology was associated with hallucinations, regardless of cognitive status. However, ADNC co‐pathology in LBD was linked to a greater NPS burden in the full cohort but not in individuals without dementia.

**DISCUSSION:**

Lewy bodies are associated with hallucinations independent of cognitive impairment, whereas ADNC co‐pathology may contribute to NPS only when widespread and associated with cognitive dysfunction.

**Highlights:**

- Neuropathological heterogeneity is high even in clinical stages without dementia.
- Neocortical but not limbic or brainstem LBD co‐pathology is associated with hallucinations.
- LBs are associated with hallucinations independent of cognitive status.
- ADNC co‐pathology is not associated with NPSs in LBD without dementia.
- LATE co‐pathology is associated with increased risk of dementia but not NPS.
- Vascular co‐pathology is associated with increased risk of delusions in ADNC.

## 1 BACKGROUND

Almost all people with dementia experience neuropsychiatric symptoms (NPSs) during the course of disease.^1^, ^2^ Depression, anxiety, apathy, agitation, delusions, and hallucinations are particularly common and have major implications, including increased caregiver burden, worsened quality of life, accelerated cognitive decline, and early institutionalization and mortality, for patients and their families.^3^, ^4^, ^5^, ^6^ However, the relative frequency and clinical course of NPSs differ across neurodegenerative dementias.^7^, ^8^ Psychosis is often the first and most frequent NPS in dementia with Lewy bodies (DLB), whereas Alzheimer's disease (AD) is associated with a broader range of initial NPSs, which increase in severity as the disease progresses.^7^, ^9^, ^10^

Different neurodegenerative pathologies have been linked to specific NPSs: Lewy body (LB) pathology is associated with hallucinations, while neurofibrillary tangles (NFTs) are more often associated with agitation and depression.^11^, ^12^, ^13^ Disinhibition and aberrant motor behavior, hallmarks of frontotemporal dementia, may be associated with limbic‐predominant age‐related transactive response DNA‐binding protein 43 (TDP‐43) encephalopathy neuropathological change (LATE‐NC).^14^, ^15^ However, neuropathological heterogeneity is now well recognized across dementia syndromes, with most patients exhibiting comorbid neurodegenerative pathologies.^16^ These additional co‐pathologies have clinical implications, including accelerated cognitive decline and increased risk of dementia.^17^, ^18^ Cases with quadruple misfolded proteinopathies (QMP) (amyloid beta [Aβ], tau, α‐synuclein, and TDP‐43 pathologies) show the most significant cognitive impairment and highest burden of NPSs.^13^, ^19^ Several *post mortem* studies suggest mixed Alzheimer's disease neuropathological change (ADNC) and Lewy body disease (LBD) are associated with increased NPSs, particularly psychosis,^11^, ^20^, ^21^ but findings are inconsistent, likely reflecting methodological differences.^15^, ^22^, ^23^

Increasingly, it appears that NPSs arise from dysfunction within specific neural networks, with different neuropathological substrates affecting specific regions and contributing to distinct clinical phenotypes.^21^, ^24^, ^25^ Neuropathological heterogeneity and associated differences in network disruption may contribute to the highly variable trajectory of NPSs both within and between dementia subtypes.^7^ However, the impact of neuropathological heterogeneity on NPS remains poorly understood, particularly in the prodromal stages. Better characterization of the mechanisms underlying NPSs and the role of comorbid neuropathology is crucial to understanding their biological basis and facilitating novel drug discovery.^26^

More than half of individuals who develop dementia are found to have NPSs before developing dementia.^27^ Mild behavioral impairment (MBI) is defined as behavioral change for more than 6 months in the absence of dementia, although mild cognitive impairment may be present.^28^ A psychiatric‐onset prodrome is also recognized in DLB.^29^ MBI is linked to increased dementia risk, proportional to the burden and complexity of NPSs.^30^ Neuropathological changes, including deposition of Aβ, tau, and neurodegeneration, are implicated in the etiology of MBI.^31^, ^32^, ^33^ However, the impact of mixed pathologies on NPSs during the prodromal stages remains unclear. Biomarker studies suggest ADNC co‐pathology may be associated with a lower risk of hallucinations and NPSs in early clinical DLB.^23^, ^34^, ^35^ Thus, circumscribed ADNC in the early DLB may not increase NPSs, but as disease progresses and ADNC becomes more widespread, ADNC co‐pathology may contribute to a higher burden of NPSs.

The Brazilian Biobank for Aging Studies (BAS) includes participants across a range of clinical stages, including many cognitively unimpaired at death, allowing for the investigation of the clinical implications of neuropathological heterogeneity across the clinical spectrum. In this autopsy study of individuals with neuropathological diagnoses of ADNC and LBD, we examined associations between cognition and NPSs with various co‐pathologies (LATE‐NC, cerebrovascular disease [CVD], LBD, and ADNC, respectively). Analysis was conducted in both the full autopsy cohort and in a “dementia‐free” subgroup. We hypothesized that hallucinations would be primarily driven by neocortical LBD and that the impact of co‐pathology on NPSs would differ by neuropathological diagnosis and anatomical distribution. Additionally, we aimed to characterize neuropathological heterogeneity in dementia‐free individuals with ADNC and LBD and to explore how co‐pathologies contribute to NPSs in the absence of dementia.

## 2 METHODS

### 2.1 Participants

Autopsy verification is mandatory in Brazil to define the cause of death for most individuals who die of natural causes. The São Paulo Autopsy Service (SPAS) is the only morgue serving the metropolitan area of São Paulo (Brazil), and participants are recruited into the Brazil Aging Study (BAS) from this service. Participants to the BAS have the brain donated by the deceased's next‐of‐kin after death upon the signature of informed consent. Between 2004 and 2014, 16% of the total number of deaths in the city were autopsied in the BAS with a demographic composition similar to that of the overall death data from São Paulo.^11^, ^36^ All BAS protocols, the informed consent form, and procedures follow international and Brazilian regulations for research involving humans and were approved by the local and federal research committees. A detailed description of the BAS procedures can be found elsewhere.^37^

For inclusion, participants must be at least 18 years of age at death and have a next of kin with whom they had at least weekly contact in the 6 months prior to death and who is able to provide clinical information and consent to brain donation. The BAS exclusion criteria include brain tissue not suitable for neuropathological analyses (cerebrospinal fluid pH < 6.5 or major acute brain lesions) or if the clinical data provided by the informant were unreliable. For the purposes of this study, cases were recruited between 2004 and 2024, and all cases over 50 years of age with neuropathological staging for LB pathology, ADNC, and CVD were included. The current study was approved by the local ethics committee.

### 2.2 Evaluation of symptoms

**RESEARCH IN CONTEXT**

1. **Systematic review**: Neuropathological heterogeneity is common, and co‐pathology is associated with increased risk of dementia and NPSs. However, few studies have explored the neuropathological heterogeneity and the implications for the psychiatric phenotype in participants without dementia.
2. **Interpretation**: Neuropathological heterogeneity is high even among participants without dementia. LBD, particularly in the neocortical regions, is associated with increased risk of hallucinations independent of cognitive status. Comorbid ADNC in LBD was associated with NPSs only when participants with dementia were included, and this effect was observed in cases with limbic, but not neocortical, LBD. TDP‐43 co‐pathology was associated with cognitive but not psychiatric symptoms in ADNC or LBD. Distinct contributions of co‐pathologies to the psychiatric phenotype were evident, with cognitive status and anatomical distribution of pathology influencing associations.
3. **Future directions**: Longitudinal studies with in vivo biomarker characterization of neuropathological change in addition to *post mortem* analysis are needed to track the dynamic changes in neuropathology during the course of disease and the implications for the cognitive and psychiatric presentation in neurodegenerative dementia.

The deceased's clinical history was obtained at the time of *post mortem* in a semi‐structured interview with the next of kin.^37^ This interview was previously validated for *post mortem* use with high sensitivity and specificity.^38^ The clinical interview includes information regarding sociodemographics, medication use, the Neuropsychiatric Inventory (NPI),^39^ and cognitive evaluation with the informant section of the Clinical Dementia Rating (CDR) scale, validated in the Brazilian population.^38^, ^40^ Scores from the CDR and 12‐item NPI collected in *post mortem* informant interviews reflect the participant's status 3 months before death to avoid the influence of delirium and peri‐agonal events.^41^ The NPI evaluates 12 domains: agitation, apathy, anxiety, appetite changes, delusions, depression, disinhibition, elation, hallucinations, irritability, motor symptoms, and sleep changes. Typically, scores are calculated by multiplying frequency (1 to 4) and severity (1 to 3) for domains with any disturbance. In this study, as the median domain scores of the participants is zero, the symptom was considered present when the domain score was above zero. To reduce subjectivity and minimize informant recall bias, the total number of NPSs was calculated based on this binary presence/absence approach. Additionally, the total burden of NPSs was calculated using the NPI‐10, which includes all NPI domains except appetite and sleep – symptoms that may be more influenced by physical health comorbidities.

### 2.3 Neuropathological assessment

The BAS uses a 14‐region immunohistochemistry panel to detect neurodegeneration and universally accepted criteria to stage and diagnose cases.^42^ Immunohistochemistry was performed in the selected sections with antibodies against Aβ (4G8, 1:10.000; Signet Pathology Systems, Dedham, Massachusetts), phosphorylated tau (PHF‐1, 1:2.000; gift from Peter Davies, New York), TDP‐43 (1:500, Proteintech, Chicago, Illinois), and α‐synuclein (EQV‐1, 1:10.000; gift from Kenji Ueda, Tokyo, Japan).^37^, ^43^, ^44^ After immunostaining for phosphor‐Ser396/Ser404 tau, NFTs were scored according to Braak stage (0, I/II, III/IV, and V/VI) following conventional categorization.^45^, ^46^ Aβ pathology was scored using CERAD‐NP for the density of neuritic plaques (none, sparse, moderate, or frequent).^47^ LB neuropathology was classified using both the Lewy Braak staging (0 to VI) and the McKeith classification.^48^, ^49^ Immunohistochemistry for TDP‐43 was introduced into the protocol in 2012 and therefore is not available for the whole cohort when recruitment started in 2004.^50^ Staging for TDP‐43 in LATE‐NC distribution was present in a subset of participants (ADNC *n* = 296 64.0%; LBD *n* = 107 58.9%). Cerebrovascular lesions were assessed by gross macroscopic evaluation of the whole brain and microscopic evaluation of the 14 regions using hematoxylin‐eosin‐stained slides. The presence of lacunar infarcts was registered by topography, stage, size, and number. The diagnosis of small vessel disease (SVD) included moderate or severe arteriosclerosis/atherosclerosis and lipohyalinosis in three or more cortical regions.^51^ Cerebral amyloid angiopathy (CAA) was considered present where widespread disease was seen in three or more cortical areas.^51^

A threshold was applied to categorize each neurodegenerative pathology as present or absent. LB pathology was considered present with a neuropathological diagnosis of LBD applied if either limbic or neocortical LB were identified, according to the McKeith criteria or a LB Braak staging ≥3. In addition, separate subgroups were included for predominant neocortical, limbic, and brainstem LBD. In two cases, the anatomical localization of LB pathology was unavailable, but a binary classification of Braak stage ≥3 was given by the reporting neuropathologist. Severity of Aβ plaque burden was classified by Consortium to Establish a Registry for Alzheimer's Disease (CERAD) staging, and the presence of NFTs was classified with Braak staging. Participants were identified as having ADNC if they were identified as having both moderate or frequent amyloid plaques in CERAD staging and Braak staging ≥III for NFTs. Participants with neuropathological diagnoses of LBD were also staged for NFTs and amyloid plaques separately to explore the individual impact of these neuropathological changes. TDP‐43 was staged with the LATE‐NC criteria, and a binary classification was applied, with TDP‐43 categorized as present in LATE‐NC stages >1. CVD was classified as present if SVD, infarct, or CAA of moderate or severe severity was identified. If only CAA was present, in the absence of other vascular disease, this was also classified separately. Neuropathological diagnoses were made blinded to clinical status.

### 2.4 Clinicopathological assessment

In the current study neuropathological diagnoses are made independent of clinical phenotypes. The term ADNC describes the neuropathological diagnosis of beta‐amyloid plaques and tau pathology detailed above; LBD describes the neuropathologic diagnosis of alpha‐synucleinopathy. Individuals with ADNC with concomitant alpha‐synucleinopathy are termed ADNC+LBD. These groups are solely defined by neuropathological diagnosis irrespective of clinical presentation and may contain individuals with a range of clinical diagnoses such as AD, DLB, and Parkinson's disease dementia (PDD).

Separately, the CDR score is used to identify the stage of dementia in individuals across neuropathological diagnoses:

‐ Participants with CDR ≥ 1 are classified as having dementia
‐ Participants with CDR = 0.5 and CDR = 0 without dementia describe individuals who may be cognitively normal or with mild cognitive impairment in the context of a neuropathological diagnosis.

However, although CDR score can identify dementia, it does not identify clinical phenotypes of dementia, and so each neuropathological group (LBD, ADNC, and ADNC+LBD) may include a mix of clinical diagnoses, including DLB, AD, or PDD.

### 2.5 Statistical analysis

Participants in the BAS with neuropathological diagnoses of ADNC or LBD were identified. The demographic and clinical differences across neuropathologic groups with ADNC, LBD, and mixed ADNC+LBD are described with descriptive statistics. In further analysis, participants were grouped by neuropathological diagnosis of ADNC (*n* = 462) or LBD (*n* = 182), and participants with mixed ADNC+LBD were included in the analysis of both respective groups. The frequency of each co‐pathology (CVD, LATE‐NC, ADNC, or LBD) was reported in both the ADNC and LBD neuropathologic groups, and, where more than one co‐pathology was present (i.e., ADNC+LATE‐NC+CVD), the participant was included in each subgroup. Thus, demographic and clinical features associated with the presence of each co‐pathology in ADNC and LBD are described in Table S1, but these differences were not formally compared across groups due to the duplication of participants with more than one co‐pathology.

**TABLE 1:** Demographic and clinical features across ADNC, LBD, and mixed ADNC+LBD neuropathological groups.

	ADNC *n* = 392	LBD *n* = 112	AD+LBD *n* = 70	*P* value
Age (mean, SD)	82.9 (8.6)	81.1 (9.0)	83.4 (7.6)	0.093
Ethnicity (%)				
White	267 (69.2)	76 (69.1)	48 (68.6)	0.934
Black	44 (11.4)	10 (9.1)	9 (12.9)	
Mixed	67 (17.4)	20 (18.1)	12 (17.1)	
Other	8 (2.1)	4 (3.6)	1 (1.4)	
Female (%)	263 (68.0)	48 (43.2)	46 (65.7)	**<0.001**
Education years (mean, SD)	4.11 (3.9)	4.49 (3.5)	5.33 (4.3)	**0.006**
Cognitive status (%)				
CN (cognitively normal)	135 (34.4)	60 (53.6)	14 (20.0)	**<0.001**
MCI	40 (10.2)	13 (11.6)	6 (8.6)	
Dementia	217 (55.4)	39 (34.8)	50 (70.4)	
CDR score (mean, SD)	1.39 (1.3)	0.83 (1.2)	1.96 (1.3)	**<0.001**
Delusions (%)	97 (25.5)	17 (15.6)	23 (33.3)	**0.020**
Hallucinations (%)	94 (24.7)	24 (21.8)	28 (40.6)	**0.012**
Agitation (%)	108 (28.4)	18 (16.4)	22 (31.9)	**0.023**
Depression (%)	113 (29.7)	38 (34.6)	24 (35.3)	0.479
Anxiety (%)	104 (27.3)	30 (27.3)	18 (26.1)	0.978
Elation (%)	13 (3.4)	2 (1.8)	4 (5.8)	0.359
Apathy (%)	99 (26.1)	26 (23.6)	22 (31.9)	0.467
Disinhibition (%)	57 (15.0)	4 (3.6)	10 (14.5)	**0.006**
Irritability (%)	81 (21.3)	11 (10.0)	16 (21.2)	**0.021**
Motor symptoms (%)	61 (16.1)	10 (9.1)	15 (21.7)	0.060
Night‐time behavior (%)	112 (29.4)	34 (31.2)	25 (36.2)	0.520
NPI‐10 number (mean, SD)	2.16 (2.3)	1.61 (1.8)	2.60 (2.1)	**0.006**
NPI‐10 total score (mean, SD)	13.5 (20.0)	8.51 (11.6)	13.9 (13.2)	**0.008**

Note: Categorical variables were analyzed using the chi‐squared (*Χ* ^2^) test. Continuous variables were assessed using a one‐way ANOVA for normally distributed data, while non‐normally distributed variables were analyzed with the Kruskal–Wallis test. Statistically significant results (*p* < 0.05) are highlighted in bold.

Abbreviation: ADNC, Alzheimer's disease neuropathological change; AD, Alzheimer's disease; LBD, Lewy body disease; NPI, Neuropsychiatric Inventory.

In both the ADNC and LBD groups, 96% of the data was complete. Little's missing completely at random test was non‐significant (*X* ^2 ^= 66.8, *p* = 0.38), suggesting the remaining 4% of data was most likely missing completely at random. In *n* = 7 participants with neuropathological data, no clinical or demographic details were reported, and given less than 5% of data was missing completely at random, complete case analysis was employed.^52^

Logistic regression was performed to evaluate the odds of the most common NPS with each co‐pathology in participants with neuropathological diagnoses of ADNC and LBD. Models are included for CAA, CVD, LATE‐NC, Braak staging of NFT, density of amyloid plaques, LBD, and ADNC co‐pathology, respectively, to explore the association between these individual neuropathological substrates and NPS. Subgroup analyses of LBD cases were also conducted based on the anatomical distribution of Lewy body pathology, categorized as neocortical, limbic, or brainstem‐predominant. Hallucinations, delusions, apathy, depression, anxiety, and agitation were included as outcomes due to their high frequency and clinical significance in DLB and AD. Models were adjusted for age, sex, ethnicity, and education. Additionally, to account for the co‐localization of neuropathological changes, the model for each neuropathology was adjusted for other neuropathological changes. Robust estimation of standard errors was used to adjust for potential heteroscedasticity, and results were presented as odds ratios. An ordinal logistic regression model was used for CDR score as the dependent variable, and a negative binomial regression model was used where the dependent variable was the number of NPS present due to overdispersion of the data. Given the exploratory nature of our study and the close correlation between NPI subscores, we used the Benjamini‐Hochberg procedure to control for the false discovery rate for the associations between NPS and each neuropathological change. This was used as a less stringent alternative to the Bonferroni correction with the false discovery rate (*Q*) set at 0.05.

We also evaluated the association between NPS and various co‐pathologies in individuals with neuropathological diagnoses of ADNC (*n* = 205) and LBD (*n* = 93) who were dementia‐free at death (CDR < 1). Logistic regression models were used to assess the odds of NPS associated with each co‐pathology in both dementia‐free ADNC and LBD cohorts. Subgroup analyses by LBD distribution (neocortical, limbic, or brainstem) were not conducted in these dementia‐free participants due to insufficient sample sizes within each subgroup. Similarly, LATE‐NC and CAA were excluded from models in the dementia‐free LBD cohort due to their low prevalence. Statistical analyses were conducted using STATA, version 18.5.

## 3 RESULTS

### 3.1 Demographic and clinical characteristics across ADNC and LBD groups

Participants with neuropathological diagnoses of ADNC, LBD, and mixed ADNC+LBD were similarly aged with similar ethnic composition (see Table 1 for detailed demographic and clinical features). Females are overrepresented in the ADNC and mixed ADNC+LBD groups relative to LBD (ADNC 68.0%; LBD 43.2%; ADNC+LBD 65.7%, *p* < 0.001). The mean years of education was low across all neuropathological groups but was significantly lower in the ADNC than ADNC+LBD group (*p* = 0.006; see Table 1). Mixed ADNC+LBD neuropathology was associated with a significantly higher prevalence of dementia than LBD without ADNC co‐pathology (*p* < 0.001), and the total score on the NPI was the highest in mixed ADNC+LBD (*p* = 0.006, see Table 1).

### 3.2 Co‐pathology in participants with neuropathological diagnosis of ADNC

**FIGURE 1:**
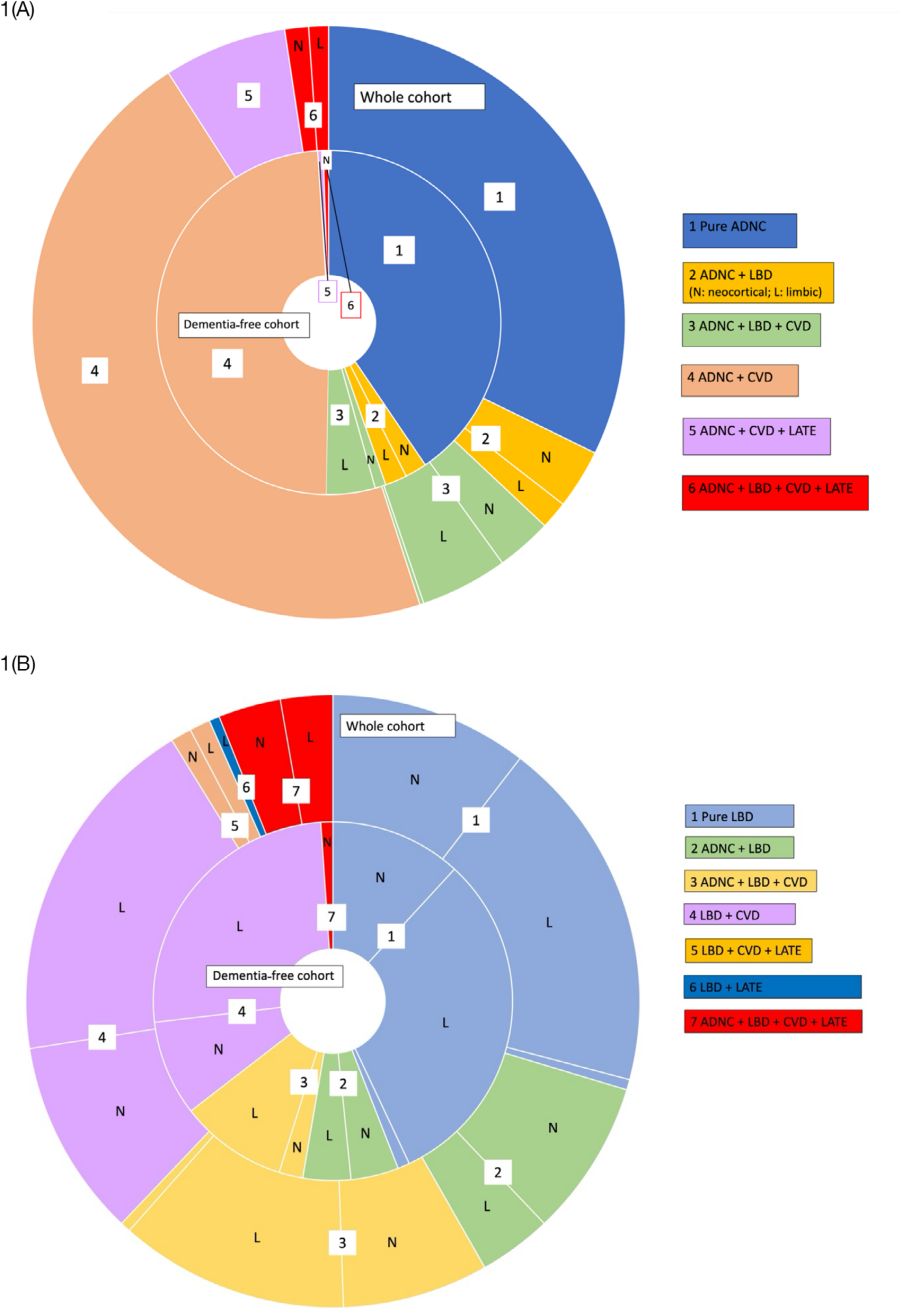
Neuropathological heterogeneity of participants with (A) ADNC and (B) LBD. (A) 1: Pure ADNC, 2: ADNC + LBD, 3: ADNC + LBD + CVD, 4: ADNC + CVD, 5: ADNC + CVD + LATE, 6: QMP: ADNC + LBD + CVD + LATE. (B) 1: Pure LBD, 2: ADNC + LBD, 3: ADNC + LBD + CVD, 4: LBD + CVD, 5: LBD + CVD + LATE, 6: LBD + LATE 7: QMP: ADNC + LBD + CVD + LATE. N: neocortical LBD, L: limbic LBD. The outer ring shows the neuropathological composition of all participants with ADNC (*n* = 462) or LBD (*n* = 182), and the inner ring includes only participants without dementia with ADNC with CDR <1 (*n* = 195) or LBD with CDR <1 (*n* = 93). TDP‐43 is only reported in *n* = 296 of the ADNC cohort and *n* = 107 of the LBD cohort. ADNC, Alzheimer's disease neuropathological change; AD, Alzheimer's disease; CDR, Clinical Dementia Rating; CAA, cerebral amyloid angiopathy; CVD, cerebrovascular disease; LBD, Lewy body disease; TDP‐43, transactive response DNA‐binding protein 43; QMP, quadruple misfolded proteinopathy.

There was marked neuropathological heterogeneity in participants with a neuropathological diagnosis of ADNC (see Figure 1A). Vascular co‐pathology was common, occurring in 63.0% of participants with ADNC; limbic or neocortical LBD co‐pathology was reported in 15.4% (neocortical LBD; *n* = 35 (50%); limbic LBD; *n* = 34 (48.6%)), LATE‐NC was described in 12.7%, while 32.5% had pure ADNC with no other neurodegenerative change (Figure 1A). An additional *n* = 9 (2.0%) of participants with ADNC had brainstem‐predominant LBD co‐pathology, but these cases were not included in the overall LBD pathological classification because brainstem LBD has rarely been associated with clinical dementia.^53^

The clinical characteristics associated with the presence of each co‐pathology in ADNC are described in Table S1. As individuals with multiple co‐pathologies were included in more than one subgroup, no formal comparisons across groups were conducted. However, among participants with ADNC, those with ‘pure ADNC’ were generally younger (mean age 81.1 ± 7.7 years), with a lower prevalence of dementia (46.9%), fewer NPS (mean 2.37 ± 2.5 each), and a lower NPI‐10 total score (mean 11.6 ± 17.0) compared to those with ADNC and additional co‐pathologies (CVD, LATE‐NC, or LBD). The most frequently reported NPS varied in ADNC subgroups according to co‐pathology: in pure ADNC, depression (30.6%) and anxiety (31.3%) were most common; in ADNC+LBD, hallucinations (40.6%) and depression (35.3%); in ADNC+LATE‐NC, agitation (42.9%) and delusions (45.7%); in ADNC+CVD, hallucinations (30.5%) and delusions (31.9%). The burden and nature of cognitive and neuropsychiatric symptoms also differed by the anatomical distribution of LBD pathology. ADNC with neocortical LBD co‐pathology was associated with a higher frequency of dementia, hallucinations, delusions, and overall NPS burden, whereas brainstem‐predominant LBD was linked to the lowest prevalence of dementia and NPS. This data is presented in Table S1.

### 3.3 Co‐pathology in participants with LBD

The neurodegenerative heterogeneity in participants with LBD is illustrated in Figure 1B and Table S1. The majority of participants with LBD (70.2%) had additional co‐pathologies; ADNC was present in 38.5% of participants, LATE‐NC was present in 15.0%, and CVD was found in 57.7% of patients with LBD. Across the whole cohort with LBD 41.2% (*n* = 75) had neocortical LBD, while 57.7% (*n* = 105) had limbic‐predominant LBD. Participants with limbic and neocortical LBD were demographically similar, but those with neocortical LBD had a higher prevalence of dementia, hallucinations, delusions, and overall burden of NPS (see Table 2). The prevalence of each co‐pathology (ADNC, LATE‐NC, CVD, and CAA) was not significantly different across participants with neocortical or limbic LBD.

**TABLE 2:** Comparison of demographic and clinical features across participants with neocortical and limbic LBD.

Clinical and demographic characteristics	Neocortical LBD (*n* = 75)	Limbic LBD (*n* = 105)	*P* value
Age (mean, SD)	82.1 (8.2)	82.1 (8.5)	0.983
Female (*n*, %)	42 (56.8)	51 (48.6)	0.280
Education years (mean, SD)	4.49 (3.8)	4.88 (3.6)	0.492
Ethnicity (*n*, %)			
White	48 (64.9)	74 (71.2)	0.355
Black	11 (14.9)	8 (7.7)	
Mixed	12 (16.2)	10 (19.2)	
Other	3 (4.1)	2 (1.9)	
Cognitive status (*n*, %)			
CN	18 (24.0)	55 (52.4)	**<0.001**
MCI	8 (10.7)	11 (10.5)	
Dementia	49 (65.3)	39 (37.1)	
Neuropsychiatric symptoms (n, %)			
Delusions	24 (33.3)	15 (14.4)	**0.003**
Hallucinations	33 (45.2)	18 (17.3)	**<0.001**
Depression	25 (34.3)	36 (35.0)	0.923
Agitation	20 (27.4)	20 (19.2)	0.201
Apathy	25 (34.3)	22 (21.4)	0.052
Anxiety	21 (28.8)	25 (24.0)	0.480
Number of NPSs in NPI‐10	2.54 (2.2)	1.59 (1.8)	**0.003**
Prevalence of co‐pathology (*n*, %)			
ADNC	35 (46.7)	34 (32.4)	0.052
CVD	41 (54.7)	63 (60.0)	0.475
CAA	17 (22.7)	18 (17.1)	0.356
TDP‐43 LATE‐NC	8 (19.5)	8 (12.3)	0.313
Pure LBD (no co‐pathology)	19 (25.3)	34 (32.7)	0.287

Note: Categorical variables were analyzed using the chi‐squared (*Χ* ^2^) test. Continuous variables were assessed using a one‐way ANOVA for normally distributed data, while non‐normally distributed variables were analyzed with the Kruskal–Wallis test. Statistically significant results (*p* < 0.05) are highlighted in bold.

Abbreviation: ADNC, Alzheimer's disease neuropathological change; CAA, cerebral amyloid angiopathy; CN, cognitively normal; CVD, cerebrovascular disease; LBD, Lewy body disease; MCI, mild cognitive impairment; NPS, neuropsychiatric symptom; TDP‐43 LATE‐NC, limbic‐predominant age‐related transactive response DNA‐binding protein 43 encephalopathy neuropathological change.

The clinical and demographic features associated with the presence of each co‐pathology (ADNC, LATE‐NC, CVD) in LBD are reported in Table S1. Pure LBD, without concomitant co‐pathology, was less common in females with LBD. Participants with ‘pure LBD’ were overall younger (79.1 + 7.3 years), with the lower prevalence of dementia (24.1%), fewer NPS in the NPI‐10 (1.23 + 1.3 each), and lower total score on the NPI‐10 (5.60 + 7.0) than participants with additional co‐pathology in LBD (ADNC, LATE‐NC, or CVD, respectively; see Table S1). Depression, apathy, hallucinations, and delusions were common in participants with LBD, irrespective of the type of co‐pathology present (see Table S1).

### 3.4 Association of co‐pathologies in ADNC with cognitive and NPS

**FIGURE 2:**
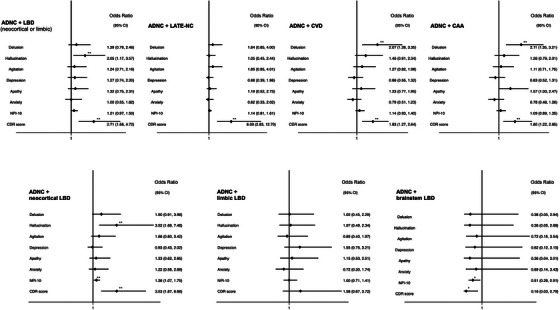
Forest plots of association between individual NPSs and each additional co‐pathology in participants with ADNC. Each NPS modeled in logistic regression adjusted for age, sex, ethnicity, education, and the presence of the other co‐pathologies. CDR modeled with ordered logistic regression, and number of NPSs modeled in negative binomial regression with incidence rate ratio reported. The odds of NPS in ADNC were also modeled with each regional subtype of LBD co‐pathology (neocortical, limbic‐predominant and brainstem‐predominant). *p < 0.05, **significance following false discovery correction. CAA, cerebral amyloid angiopathy; CVD, cerebrovascular disease; LBD, Lewy body disease; TDP‐43 LATE‐NC, limbic‐predominant age‐related TDP‐43 encephalopathy.

In logistic regression adjusted for age, sex, ethnicity, education, and LB pathology, the presence of CVD or CAA was associated with an increased risk of delusions in participants with ADNC (CVD OR = 2.07 [95% CI 1.28–3.35], *p* = 0.003; CAA OR = 2.11 [95% CI 1.35–3.21], *p* = 0.001) compared to those without CVD. The presence of LB co‐pathology in the limbic or neocortical regions was associated with increased risk of hallucinations in ADNC in logistic regression adjusted for age, sex, ethnicity, education, and CVD (OR = 2.05 [95% CI 1.17–3.57], *p* = 0.011). However, when analyzed separately by anatomical distribution, only neocortical LBD co‐pathology was associated with increased risk of hallucinations in ADNC (OR = 3.55 [95% CI 1.68–7.52], *p* = 0.001), while limbic (OR = 0.92 [95% CI 0.39–2.17], *p* = 0.844) and brainstem LB pathology were not (OR = 0.36 [95% CI 0.05–2.68], *p* = 0.319). Neocortical LBD co‐pathology was also associated with increased overall burden of NPS in ADNC (OR = 1.43 [95% CI 1.13–1.80], *p* = 0.003). The presence of any co‐pathology (LBD, LATE‐NC, CVD, or CAA) in ADNC was associated with significantly increased risk of higher CDR scores (see Figure 2) but when separated by distribution, neocortical LBD, but not limbic or brainstem LBD, was associated with higher CDR scores in ADNC (OR 3.52 [95% CI 1.87–6.63], *p* < 0.001) (see Figure 2).

### 3.5 Association between cognitive and neuropsychiatric symptoms and co‐pathology in LBD

**FIGURE 3:**
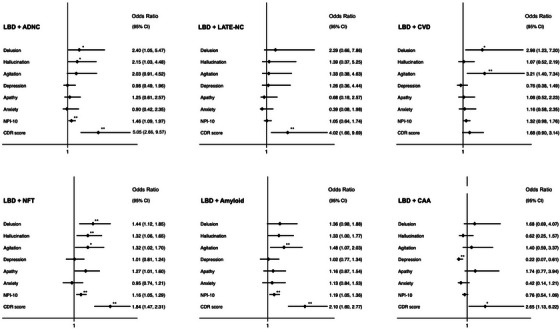
Forest plots of association between individual NPSs and each additional co‐pathology in participants with LBD. Each NPS modeled in logistic regression adjusted for age, sex, ethnicity, education, and the presence of the other co‐pathologies. CDR modeled with ordered logistic regression, and number of NPSs modeled in negative binomial regression with incidence rate ratio reported. ADNC, Alzheimer's disease neuropathological change; CAA, cerebral amyloid angiopathy; CVD, cerebrovascular disease; NFT, neurofibrillary tangles staged by Braak criteria; Amyloid, density of plaques staged by CERAD criteria; TDP‐43 LATE‐NC, limbic‐predominant age‐related TDP‐43 encephalopathy. *p < 0.05, **significance following false discovery correction.

In logistic regression adjusted for age, sex, ethnicity, education, and CVD, the presence of ADNC co‐pathology in LBD was associated with greater overall number of NPS (OR 1.46 [1.09–1.97], *p* = 0.012; Figure 3). ADNC co‐pathology in LBD was also associated with increased risk of hallucinations and delusions, but the associations did not meet significance once corrected for the false discovery rate (hallucinations OR = 2.15, [95% CI 1.03–4.48], *p* = 0.042; delusions OR = 2.40, [95% CI 1.05–5.47], *p* = 0.038). However, increasing Braak stage of NFT was independently associated with an increased risk of delusions, hallucinations, and the overall burden of NPS in LBD. Similarly, greater amyloid plaque burden, as defined by CERAD staging, was also independently associated with agitation and the total burden of NPS in LBD. Vascular co‐pathology was associated with increased risk of delusions and agitation in LBD. In addition, ADNC (including the stage of NFT and density of amyloid plaque independently) and LATE co‐pathology were associated with increased CDR scores in LBD (Figure 3).

When the impact of ADNC co‐pathology was examined separately in participants with limbic versus neocortical LBD, distinct patterns emerged. In limbic LBD, ADNC co‐pathology was associated with significantly increased risk of delusions (OR 6.47 [1.45–28.9], *p* = 0.014), hallucinations (OR 3.07 [1.03–9.17], *p* = 0.044), and overall NPS burden (IRR 1.66 [1.05–2.63], *p* = 0.030). There were no significant associations with ADNC co‐pathology and apathy, agitation, or depression in limbic LBD. In contrast, in cases with neocortical LBD, ADNC co‐pathology was not associated with increased burden of NPS (IRR 1.21 [0.84–1.73], *p* = 0.298) or with any individual NPS. However, in both neocortical and limbic LBD, ADNC co‐pathology was associated with increased risk of dementia (neocortical OR 3.65 [1.39–9.60], *p* = 0.009; limbic OR 6.29 [2.23–17.8], *p* = 0.001).

### 3.6 Demographic and clinical characteristics of participants with ADNC and LBD without dementia

There was a range of clinical stages across the neuropathological diagnoses of ADNC and LBD. However, a significantly higher proportion of the participants with a neuropathological diagnosis of LBD were dementia‐free at the time of death than those with a neuropathological diagnosis of ADNC or mixed ADNC+LBD (see Tables 1 and S1). In participants at the dementia‐free stages of disease, females remained underrepresented in participants with neuropathological diagnosis of LBD relative to ADNC and mixed ADNC+LBD (*p* = 0.009) (Table S2). However, there was no difference in the overall burden of NPS across AD, LBD, and mixed AD+LBD groups with neuropathological diagnoses without dementia, but hallucinations were significantly more frequent in participants without dementia with LBD or mixed ADNC+LBD than ADNC without LBD co‐pathology (see Table S2).

### 3.7 Co‐pathology in ADNC without dementia

Under half of participants with a neuropathological diagnosis of ADNC did not have dementia (CDR < 1; *n* = 205 (45.4%)). There was a higher proportion of participants with pure ADNC (38.5%) in participants without dementia than across the whole cohort. In participants with ADNC without dementia, vascular co‐pathology was most common (55.1%), and 9.8% had LBD co‐pathology (35% neocortical; 55% limbic predominant) (see inner circle of Figure 1A; Table S3).

### 3.8 Co‐pathology in LBD without dementia

In participants with a neuropathological diagnosis of LBD, 51.4% (*n* = 93) did not have dementia, *n* = 26 (26.9%) with neocortical LBD and *n* = 66 (68.8%) with limbic LBD. Pure LBD was more common in participants without dementia than across the whole cohort (44.1%), and comorbid ADNC was found in 21.5% of participants with LBD without dementia (see inner circle of Figure 1B; Table S3).

Although pure disease was more common in participants with neuropathological diagnoses of ADNC and LBD without dementia, the overall neuropathological heterogeneity remained significant at this stage (shown in Figure 1).

### 3.9 Association between co‐pathology and NPS in ADNC without dementia

**FIGURE 4:**
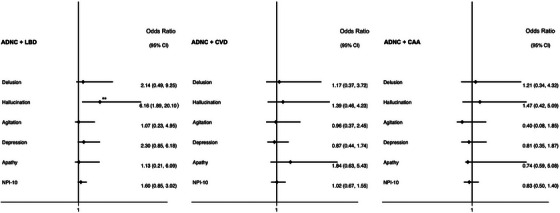
Forest plots of association between individual NPSs and each co‐pathology in participants with ADNC without dementia. Each NPS modeled in logistic regression adjusted for age, sex, ethnicity, education, and the presence of the other co‐pathologies. NPI‐10: total number of NPSs modeled in negative binomial regression with incidence rate ratio reported. CAA, cerebral amyloid angiopathy; LBD, Lewy body disease in neocortical or limbic regions. *p < 0.05, **significance following false discovery correction.

In participants without dementia with neuropathological diagnoses of ADNC, the presence of limbic or neocortical LB co‐pathology was associated with an increased risk of hallucinations (OR 5.78 [95% CI 1.82–18.3], *p* = 0.003) in adjusted logistic regression. CVD was not associated with changes in NPS in participants with ADNC without dementia (see Figure 4).

### 3.10 Association between co‐pathology and NPS in LBD without dementia

**FIGURE 5:**
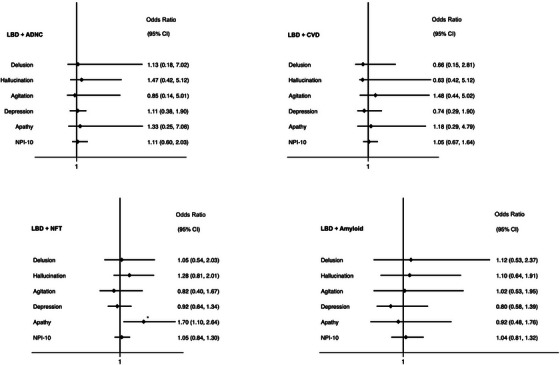
Forest plots of association between individual NPSs and each co‐pathology in participants with LBD without dementia. Each NPS modeled in logistic regression adjusted for age, sex, ethnicity, education, and the presence of the other co‐pathologies. Number of NPSs modeled in negative binomial regression with incidence rate ratio reported. ADNC, Alzheimer's disease neuropathological change; NFT, neurofibrillary tangles. *p < 0.05.

In the participants with LBD without dementia, the presence of ADNC or vascular changes was not associated with an increased risk of NPS. However, increased Braak stages of NFT were associated with an increased risk of apathy in dementia‐free LBD (Figure 5).

## 4 DISCUSSION

In this single‐center autopsy study of 574 cases with neuropathological diagnoses of ADNC or LBD, we examined how co‐pathologies relate to NPS in participants with and without dementia. As in prior studies, co‐pathology was common in both ADNC and LBD and associated with greater cognitive impairment.^16^, ^54^ We also identified distinct associations between NPS and co‐pathologies across different stages of clinical disease in ADNC and LBD.

Previous studies have explored the neuropsychiatric phenotype associated with neuropathological change, but results have been inconsistent, likely reflecting differences in study design, disease stage, and pathological distribution.^8^ *Post mortem* studies often report that mixed ADNC and LBD is associated with an increased burden of NPS, particularly psychosis,^13^, ^20^ whereas in vivo biomarker studies in earlier disease suggest hallucinations and NPS are more prominent in ‘pure’ LBD, in the absence of ADNC.^23^, ^35^, ^55^

In the current study, ADNC co‐pathology in LBD was associated with increased burden of NPS. More widespread NFTs in LBD were also independently associated with increased delusions, hallucinations, and overall NPS, while increasing density of amyloid plaques was associated with agitation and global NPS. However, the impact of ADNC co‐pathology varied according to the distribution of LBD. ADNC co‐pathology was associated with increased odds of delusions, hallucinations, and burden of NPS in limbic but not neocortical LBD. This suggests that it is the pattern of cortical involvement, rather than inherent disease‐specific pathology, which is a key determinant of NPS. This may also contribute to differences between *post mortem* and in vivo studies, with the former more likely to include cases with limbic or brainstem LBD, which may not produce sufficient clinical symptoms to meet diagnostic criteria for DLB in vivo. Furthermore, if degree of cortical spread is a key factor driving the emergence of NPS, this may also explain why the QMP phenotype—characterized by co‐occurring ADNC, LBD, and LATE‐NC—is associated with the greatest NPS burden.^13^

Among dementia‐free participants with LBD, the presence of co‐pathology (ADNC, CVD or LATE‐NC), was not associated with NPS suggesting that α‐synuclein may primarily drive early‐stage NPS. This supports the recent in vivo studies suggesting ADNC co‐pathology is associated with fewer NPS and hallucinations in early LBD.^23^, ^34^, ^56^ Additionally, in our population‐based cohort, individuals with LBD were less likely than those with ADNC to have dementia at death, suggesting the progression to clinical dementia in LBD may require more extensive α‐synucleinopathy or synaptic dysfunction—which may simultaneously drive the emergence of core symptoms such as visual hallucinations.

These findings suggest that the NPS phenotype in mixed AD+LBD is dynamic and disease‐stage dependent. In the dementia‐free stages of LBD, ADNC co‐pathology does not appear to contribute significantly to NPS, but in the later stages of LBD—when cognitive impairment and network dysfunction are more widespread—ADNC co‐pathology, and particularly tau pathology, may increase the burden of NPS, including hallucinations and delusions.

In contrast, in ADNC, LBD co‐pathology was associated with hallucinations even in the absence of dementia, suggesting LBD may drive hallucinations independent of cognitive dysfunction. Lewy bodies in the limbic and neocortical regions have previously been implicated in the etiology of visual hallucinations irrespective of the overarching neurodegenerative diagnosis,^57^, ^58^, ^59^, ^60^ but in the current study, neocortical—but not limbic—LBD co‐pathology was associated with hallucinations in the context of ADNC.

Current models suggest visual hallucinations arise from dysfunctional processing of visual sensory information combined with overweighted top‐down processing of sensory input,^61^, ^62^ integrated across networks throughout the cortical hierarchy.^63^ Although the neurobiological correlates of such models remain undefined, it seems likely that the selective topography of LBD disrupts these networks early, independent of cognition from the dementia‐free stages of disease, whereas ADNC may only be implicated as it becomes more widespread in the dementia stages of disease. Supporting this, *post mortem* studies suggest visual hallucinations are discriminatory for LBD in the early stages, but in later disease, visual hallucinations are equally common in ADNC.^64^ This suggests the association between ADNC and NPS is intrinsically linked to cognitive status while neocortical LB pathology may be associated with hallucinations independent of cognition. This may also account for the greater increase in hallucinations and overall NPS burden observed during the dementia stages of ADNC in the current cohort, in contrast to LBD, where the frequency of NPS and hallucinations remained high across all clinical stages. Furthermore, while MBI is highly predictive of prognosis and incident dementia in MBI,^30^ NPS have not been associated with cognitive decline in prodromal DLB.^65^

In AD, apathy has previously been associated with the spread of Aβ and tau pathology, independent of cognitive decline.^31^, ^66^, ^67^, ^68^ In the present study, apathy was similarly associated with the burden of NFT co‐pathology in the dementia‐free stages of LBD. Recent tau‐positron emission tomography imaging suggests tau deposition in the parietal and temporal regions is associated with apathy in the early disease, even in the absence of cognitive impairment.^68^ In the later stages of clinical AD, apathy is associated with tau deposition in the frontal regions, including the anterior cingulate cortex, and correlates with cognitive dysfunction.^69^, ^70^ Cortical amyloid has also been associated with apathy and may potentiate the effects of tau with resultant higher levels of apathy.^68^ These findings suggest tau pathology may contribute to apathy from the earliest stages of disease, prior to any emerging cognitive impairment and independent of the neuropathological diagnosis.

In ADNC, vascular co‐pathology was associated with increased risk of both delusions and dementia. This aligns with previous studies linking CVD, including SVD, arteriosclerosis, and CAA, with psychosis in AD.^22^, ^71^, ^72^, ^73^, ^74^ White matter lesions and subcortical infarcts may increase susceptibility to delusions by disrupting structural connectivity across widespread networks. Although less studied in DLB, CVD is increasingly recognized as both prevalent and clinically relevant.^75^, ^76^, ^77^, ^78^ However, in the current study, the association between CVD and CAA co‐pathology and delusions and dementia in LBD did not remain significant after correction for multiple comparisons, suggesting a weaker relationship than in ADNC. Additionally, white matter hyperintensities in DLB have previously been associated with greater medial temporal lobe atrophy, in an effect dependent on Aβ co‐pathology. This suggests the association between WMH and DLB is to some extent mediated by amyloid.^79^

Consistent with previous studies, LATE‐NC co‐pathology was not associated with greater burden of NPS in ADNC or LBD after adjusting for other co‐pathologies.^80^ However, LATE‐NC was staged in a smaller cohort of participants (*n* = 286) and was present in *n* = 36 (12.7%) participants with ADNC and *n* = 16 (15.0%) with LBD, and therefore, the current study may have been underpowered to detect associations between TDP‐43 co‐pathology and NPS.

### 4.1 Strengths and limitations

The population‐based design of the BAS reduces selection bias and enables inclusion of a wide range of cognitive outcomes at death in a diverse population broadly representative of mortality data from São Paulo. However, some selection bias remains, including characteristics relating to the consenting informant and the exclusion of traumatic deaths. Nevertheless, we were able to select a large group with neuropathological diagnoses of ADNC and LBD without dementia, allowing the characterization of associations between co‐pathology and NPSs independent of cognition. However, as with all cross‐sectional *post mortem* studies, the findings reported in this study are inherently correlational, and no causation or longitudinal relationships can be inferred.

There are several additional limitations to this study. First, the NPI was completed after death in a semi‐structured interview with the next of kin for the period 3 months prior to death. This timeframe was used to reduce the influence of NPSs common near the time of death, such as those present during delirium episodes, but introduces potential recall bias. However, the semi‐structured interview with informants has been validated for both the clinical dementia ratings and assessment of NPSs,^38^, ^81^ and to reduce the influence of recall bias, informants are required to have at least weekly contact with the deceased prior to death. Additionally, to reduce potential subjectivity from informants, all NPSs are included on a binary basis. However, given the cross‐sectional nature of the study, there remains a risk of confounding, such as hallucinations potentially being related to delirium rather than representing a core feature of LBD. Future studies should aim to incorporate longitudinal data to distinguish transient symptoms from those that are disease‐specific.

Secondly, although we identified participants with limbic and neocortical LBD, due to the smaller sample size of these subgroups, we were not able to conduct this subgroup analysis in the dementia‐free patient populations. Additionally, although clinical severity was staged using the CDR score, the absence of clinical diagnoses in this study is a significant limitation. Without clinical diagnostic information, we were unable to distinguish DLB from PDD or, among participants without dementia, between PDD and asymptomatic individuals with LBD. As a result, we could not examine how relationships between neuropathology and NPS might differ across these clinical contexts, particularly relevant in participants without dementia, where the implications of a diagnosis of Parkinson's disease differ substantially from incidental LBD. The lack of clinical diagnostic data also precluded assessment of concordance between clinical syndromes and underlying pathology and the impact of mixed neuropathological disease. Finally, as discussed, LATE‐NC was not staged in all participants (*n* = 359, 62.4%, staged in all cases after 2012, when this staging was incorporated into the protocol), and LATE‐NC was uncommon in participants without dementia. Therefore, while we adjusted for co‐occurring neuropathologies to account for the clinical implications of co‐localization of neurodegenerative pathologies, we did not adjust for LATE‐NC. Furthermore, the reduced numbers with staging for LATE‐NC reduced the statistical power to detect associations between NPS and TDP‐43 as a co‐pathology in ADNC and LBD.

## 5 CONCLUSIONS

This study highlights the importance of co‐pathologies for NPSs in ADNC and LBD and differences across the clinical stages of disease. LBD was associated with hallucinations in the absence of dementia, while ADNC co‐pathology may be associated with greater burden of NPSs only in later disease stages. This suggests specific neuropathologies disrupt distributed brain networks at different points along the disease trajectory, contributing to the emergence of NPSs in a dynamic, stage‐dependent manner. This highlights the importance of a precision medicine approach to managing the inherent neuropathological heterogeneity common in patients.

## CONFLICT OF INTEREST STATEMENT

Dr. Aarsland has received research support and/or honoraria from Astra‐Zeneca, H. Lundbeck, Novartis Pharmaceuticals, Evonik, Roche Diagnostics, GE Health, and Sanofi and served as a paid consultant for H. Lundbeck, Eisai, Heptares, Eli Lilly, Enterin, Acadia, EIP Pharma, Biogen, Takeda, and BioArctic. Dr. Grinberg reported personal fees from Guidepoint (consulting), Otsuka (educational event), Medscape, and Weill Neurohub outside the submitted work. The other authors have no disclosures to report. Author disclosures are available in the Supporting Information.

## CONSENT STATEMENT

In all *post mortem* cases the next of kin provided signed informed consent.

## Supporting information



Supporting Information

Supporting Information

## Data Availability

The data that support the findings of this study are available from the corresponding author upon reasonable request.
