# Supplementary material for: Co‐pathology in Alzheimer's disease and Lewy body disease and its association with neuropsychiatric symptoms
Source: Alzheimers Dement. 2025 Sep 15;21(9):e70693. doi: 10.1002/alz.70693 (PMC12434609; doi:10.1002/alz.70693)
Supplement: Supplementary file 2 — Supporting Information [file ALZ-21-e70693-s001.docx]

|  | |  |  | Alzheimer’s disease neuropathological change | | | | | | | | | Neocortical or limbic Lewy body pathology | | | | | |
| --- | --- | --- | --- | --- | --- | --- | --- | --- | --- | --- | --- | --- | --- | --- | --- | --- | --- | --- |
|  | | Across the whole cohort with ADNC (n=462) | Across the cohort with LBD (n=182)  75 nLBD,  105 lLBD,  2 unclassified | Co-pathology in participants ADNC | | | | | | | | | Co-pathology in participants with neocortical (nLBD) or limbic (lLBD) LBD | | | | | |
|  |  |  |  | Only ADNC (n=149; 32.5%) | LBD  (n=70; 15.1) | LBD by anatomical distribution | | | | LATE-NC > stage 1 (n=36; 12.7%) | CAA (n=136; 29.6%) | CVD (n=291; 63.0%) | Only LBD (n=54, 29.7%)  19 nLBD  35 lLBD  1 unclassified | ADNC  (n=70, 38.5%)  35 nLBD  34 lLBD | LATE-NC >stage 1 (n=16, 15.0%)  8 nLBD  8 lLBD | CAA (n=36, 19.8 %)  17 nLBD  18 lLBD | | CVD (n=105, 57.7%)  41 nLBD  63 lLBD |
|  |  |  |  |  |  | Neocortical  n=35; 7.7% | | Limbic  n=34 6.8% | Brainstem  n=9 2.0% |  |  |  |  |  |  |  |  |  |
| Demographic information (n, %) | | | | | | | | | | | | | | | | | | |
| Age (mean, SD) | | 83.1 (8.4) | 82.1 (8.1) | 81.1 (7.7) | 83.7 (7.7) | 83.4 (8.1) | 83.8 (7.4) | | 84.7 (4.9) | 85.8 (8.3) | 83.9 (9.3) | 84.1 (8.8) | 79.1 (7.3) | 83.7 (7.7) | 86.2 (7.1) | | 83.9 (10.2) | 83.6 (8.9) |
| Ethnicity | White | 315 (69.1) | 124 (68.9) | 104 (71.2) | 48 (68.6) | 22 (62.9) | 25 (73.5) | | 8 (88.9) | 26 (74.3) | 93 (69.4) | 198 (69.2) | 39 (72.2) | 48 (68.6) | 11 (73.3) | | 28 (77.8) | 73 (70.2) |
|  | Black | 53 (11.6) | 19 (10.6) | 19 (13.0) | 9 (12.9) | 7 (20.0) | 2 (5.9) | | 0 | 3 (8.6) | 13 (9.7) | 30 (10.5) | 3 (5.6) | 9 (12.9) | 3 (20.0) | | 3 (8.3) | 12 (11.5) |
|  | Mixed | 79 (17.3) | 32 (17.8) | 21 (14.4) | 12 (17.1) | 5 (14.3) | 7 (20.6) | | 1 (11.1) | 5 (14.3) | 28 (20.2) | 51 (17.8) | 9 (16.7) | 12 (17.1) | 1 (6.7) | | 5 (13.9) | 17 (16.4) |
|  | Other | 9 (2.0) | 5 (2.8) | 2 (1.4) | 1 (1.4) | 1 (2.9) | 0 | | 0 | 1 (2.9) | 1 (0.8) | 7 (2.5) | 3 (5.6) | 1 (1.4) | - | | - | 2 (1.9) |
| Female | | 309 (67.6) | 94 (51.9) | 99 (67.8) | 46 (65.7) | 24 (68.6) | 22 (64.7) | | 6 (66.7) | 28 (80.0) | 88 (65.7) | 195 (67.9) | 26 (48.2) | 46 (65.7) | 12 (80) | | 24 (66.7) | 54 (51.9) |
| Education years | | 4.29 (4.0) | 4.76 (3.7) | 3.35 (3.0) | 5.41 (4.4) | 5.0 (4.6) | 5.65 (4.1) | | 4.78 (6.7) | 4.60 (4.6) | 4.59 (4.1) | 4.72 (4.4) | 3.78 (2.5) | 5.41 (4.4) | 5.27 (3.9) | | 5.67 (4.3) | 5.20 (4.1) |
| Cognitive and neuropsychiatric symptoms (n, %) | | | | | | | | | | | | | | | | | | |
| Cognitive status | CN (cognitively normal) | 149 (32.3) | 74 (40.7) | 59 (40.1) | 14 (20.0) | 4 (11.4) | 10 (29.4) | | 7 (77.8) | 1 (2.9) | 33 (24.6) | 84 (29.5) | 33 (61.1) | 14 (20.0) | 0 | | 6 (16.7) | 36 (34.3) |
|  | MCI | 46 (10.0) | 19 (10.4) | 19 (12.9) | 6 (8.6) | 3 (8.6) | 3 (8.8) | | 0 | 1 (2.9) | 11 (8.2) | 24 (8.4) | 8 (14.8) | 6 (8.6) | 1 (6.7) | | 2 (5.6) | 8 (7.6) |
|  | Dementia | 267 (57.8) | 89 (48.9) | 69 (46.9) | 50 (71.4) | 28 (80.0) | 21 (61.8) | | 2 (22.2) | 34 (94.4) | 90 (67.2) | 177 (62.1) | 13 (24.1) | 50 (71.4) | 15 (93.8) | | 28 (77.8) | 61 (58.1) |
| CDR score (mean, SD) | | 1.49 (1.5) | 1.27 (1.3) | 1.12 (1.2) | 2.04 (1.3) | 2.21 (1.2) | 1.84 (1.4) | | 0.44 (1.0) | 2.59 (0.8) | 1.79 (1.3) | 1.66 (1.3) | 0.61 (1.04) | 2.04 (1.3) | 2.50 (0.8) | | 2.22 (1.2) | 1.58 (1.4) |
| Delusions | | 120 (26.7) | 40 (22.5) | 25 (17.5) | 23 (33.3) | 13 (38.2) | 10 (29.4) | | 1 (11.1) | 16 (45.7) | 50 (37.9) | 90 (31.9) | 5 (7.6) | 23 (33.3) | 8 (53.3) | | 13 (37.1) | 32 (31.1) |
| Hallucinations | | 122 (27.2) | 52 (29.1) | 25 (17.5) | 28 (40.6) | 18 (52.9) | 10 (29.4) | | 1 (11.1) | 11 (31.4) | 42 (31.8) | 86 (30.5) | 10 (18.5) | 28 (40.6) | 6 (40.0) | | 11 (31.4) | 32 (31.1) |
| Agitation | | 130 (28.9) | 40 (22.4) | 39 (27.1) | 22 (31.9) | 13 (38.2) | 9 (26.5) | | 2 (22.2) | 15 (42.9) | 40 (30.3) | 85 (30.1) | 4 (7.4) | 22 (31.9) | 6 (40.0) | | 10 (28.6) | 31 (30.1) |
| Depression | | 137 (30.6) | 62 (34.8) | 44 (30.6) | 24 (35.3) | 10 (29.4) | 13 (39.4) | | 2 (22.2) | 10 (28.6) | 37 (28.2) | 83 (29.6) | 19 (35.2) | 24 (35.3) | 6 (40.0) | | 6 (17.7) | 34 (33.3) |
| Anxiety | | 122 (27.1) | 48 (26.8) | 45 (31.3) | 18 (26.1) | 10 (29.4) | 7 (20.6) | | 2 (22.2) | 7 (20.0) | 31 (23.5) | 70 (24.8) | 14 (25.9) | 18 (26.1) | 2 (13.3) | | 6 (17.7) | 28 (27.2) |
| Elation | | 17 (3.8) | 6 (3.4) | 4 (2.8) | 4 (5.8) | 2 (5.6) | 2 (5.9) | | 0 | 1 (2.9) | 3 (2.3) | 13 (4.6) | 1 (1.9) | 4 (5.8) | 0 | | 2 (5.7) | 5 (4.9) |
| Apathy | | 121 (27.0) | 48 (26.8) | 31 (21.7) | 22 (31.9) | 11 (32.4) | 10 (29.4) | | 1 (11.1) | 12 (34.3) | 45 (34.1) | 81 (28.7) | 10 (18.5) | 22 (31.9) | 4 (26.7) | | 13 (37.1) | 30 (29.1) |
| Disinhibition | | 67 (14.9) | 14 (7.8) | 21 (14.6) | 10 (14.5) | 6 (17.7) | 4 (11.8) | | 1 (11.1) | 7 (20.0) | 17 (12.9) | 43 (15.3) | 1 (1.9) | 10 (14.5) | 3 (20.0) | | 3 (8.6) | 10 (9.7) |
| Irritability | | 97 (21.6) | 27 (15.1) | 30 (20.8) | 16 (23.2) | 9 (26.5) | 7 (20.6) | | 0 | 6 (17.1) | 26 (19.7) | 61 (21.6) | 2 (3.7) | 16 (23.2) | 2 (13.3) | | 6 (17.1) | 19 (18.5) |
| Motor symptoms | | 76 (16.9) | 25 (14.0) | 19 (13.3) | 15 (21.7) | 8 (23.5) | 7 (20.6) | | 0 | 9 (25.7) | 26 (19.7) | 52 (18.4) | 4 (7.4) | 15 (21.7) | 4 (26.7) | | 6 (17.1) | 16 (15.5) |
| NPI-10 number (mean, SD) | | 2.23 (2.3) | 1.99 (2.0) | 1.99 (2.2) | 2.60 (2.1) | 2.94 (2.0) | 2.24 (2.2) | | 1.11 (1.1) | 2.69 (2.3) | 2.38 (2.4) | 2.33 (2.4) | 1.23 (1.3) | 2.60 (2.1) | 2.73 (2.2) | | 2.09 (1.9) | 2.27 (2.2) |
| NPI-10 total score (mean, SD) | | 13.6 (19.1) | 10.5 (12.5) | 11.6 (17.0) | 13.9 (13.2) | 15.3 (13.3) | 12.4 (13.1) | | 4 (3.8) | 18.8 (25.3) | 15 (19.7) | 14.5 (20.5) | 5.60 (7.0) | 13.9 (13.2) | 12.3 (10.7) | | 9.56 (10.7) | 12.1 (13.8) |

Supplementary table 1. Demographic and clinical features across ADNC and LBD neuropathological groups separated by additional co-pathologies.

Abbreviations: ADNC, Alzheimer’s disease neuropathological change; LBD, Lewy body disease in neocortical or limbic regions; LATE-NC, Limbic-predominant age-related TDP-43 encephalopathy neuropathologic change; CVD, cerebrovascular disease; CAA cerebral amyloid angiopathy. nLBD, neocortical-predominant LBD; lLBD, limbic-predominant LBD; bLBD, brainstem-predominant LBD; CN, cognitively normal; MCI, mild cognitive impairment; CDR, clinical dementia rating; NPI-10, neuropsychiatric inventory excluding appetite and sleep items.

Supplementary table 2. Demographic and clinical features across participants with neuropathological diagnoses of ADNC and LBD but without clinical dementia separated by co-pathologies.

|  | | ADNC n=175 | LBD n=73 | AD+LBD n=20 | P value |
| --- | --- | --- | --- | --- | --- |
| Age (mean, SD)^^^ | | 81.8 (9.2) | 79.4 (8.9) | 84.2 (9.8) | 0.063 |
| Ethnicity | White | *126 (72)* | *48 (65.8)* | *16 (80)* | 0.702 |
|  | Black | *17 (9.7)* | *6 (8.2)* | *2 (10.0)* |  |
|  | Mixed | *27 (15.4)* | *15 (20.6)* | *2 (10.0)* |  |
|  | Other | *5 (2.9)* | *4 (5.5)* | *0* |  |
| Female | | *102 (58.3)* | *29 (39.7)* | *14 (70.0)* | **0.009** |
| Education years | | 4.61 (4.1) | 4.3 (3.2) | 5.8 (4.4) | 0.334 |
| *Neuropsychiatric symptoms (n, %)* | | | | | |
| Delusions | | 12 (6.9) | 8 (11.0) | 3 (15.0) | 0.333 |
| Hallucinations | | 11 (6.3) | 12 (16.4) | 6 (30) | **0.001** |
| Agitation | | 16 (9.2) | 7 (9.6) | 2 (10.0) | 0.990 |
| Depression | | 42 (24.1) | 25 (34.3) | 8 (40.0) | 0.127 |
| Anxiety | | 41 (23.6) | 20 (27.4) | 6 (30.0) | 0.712 |
| Elation | | 3 (1.7) | 1 (1.4) | 2 (10.0) | 0.051 |
| Apathy | | 17 (9.8) | 9 (12.3) | 2 (10.0) | 0.834 |
| Disinhibition | | 8 (4.6) | 0 | 1 (5.0) | 0.173 |
| Irritability | | 26 (14.9) | 5 (6.9) | 1 (5.0) | 0.123 |
| Motor symptoms | | 5 (2.9) | 2 (2.7) | 0 | 0.745 |
| Night-time behaviour | | 109 (28.8) | 35 (30.4) | 4 (20.0) | 0.214 |
| NPI-10 number (mean, SD) | | 1.03 (1.6) | 1.22 (6.3) | 1.55 (2.0) | 0.112 |
| NPI-10 total score (mean, SD) | | 5.3 (12.0) | 6.3 (9.6) | 8.9 (13.1) | 0.081 |

Categorical variables were analyzed using the Chi-square (Χ²) test. Continuous variables were assessed using a one-way ANOVA for normally distributed data, while non-normally distributed variables were analyzed with the Kruskal-Wallis test. Statistically significant results (p < 0.05) are highlighted in bold

Supplementary table 3. Demographic and clinical features across participants with neuropathological diagnoses of ADNC and LBD without clinical dementia separated by co-pathologies.

The frequency of each co-pathology was reported irrespective of other neurodegenerative changes in ADNC and LBD and where more than one co-pathology was present the participant was included in both subgroups. Numbers are absolute (n) and relative (%) frequencies if not otherwise specified.

|  | | ADNC without dementia (n=205) | LBD without dementia (n=93);  26 nLBD  66 lLBD | Alzheimer’s disease neuropathological change | | | | Neocortical (n) or limbic (l) Lewy body pathology | | |
| --- | --- | --- | --- | --- | --- | --- | --- | --- | --- | --- |
|  |  |  |  | Co-pathology in participants with ADNC without dementia | | | | Co-pathology in participants with neocortical (n) or limbic (l) LBD without dementia | | |
|  |  |  |  | Pure ADNC (n=79; 38.5%) | LBD  (n=20; 9.8%)  7 nLBD  13 lLBD | CAA (n=47; 22.9%) | CVD (n=113; 55.1%) | Pure LBD (n=41, 44.1%)  11 nLBD  29 lLBD | ADNC  (n=20, 21.5%)  7 nLBD  13 lLBD | CVD (n=44, 47.3%)  11 nLBD  33 lLBD |
| Demographic information (n, %) | | | | | | | | | | |
| Age (mean, SD) | | 82.0 (9.1) | 80.4 (9.3) | 80.4 (8.0) | 84.2 (9.8) | 81.4 (11.4) | 83.2 (10.0) | 77.6 (7.0) | 84.2 (9.8) | 82.4 (11.0) |
| Ethnicity | White | 151 (73.7) | 64 (68.8) | 57 (72.2) | 16 (80.0) | 34 (72.3) | 82 (72.6) | 29 (70.7) | 16 (80.0) | 28 (63.6) |
|  | Black | 20 (9.8) | 8 (8.6) | 9 (11.4) | 2 (10.0) | 5 (10.6) | 10 (8.9) | 2 (4.9) | 2 (10.0) | 5 (11.4) |
|  | Mixed | 29 (14.2) | 17 (18.3) | 11 (13.9) | 2 (10.0) | 8 (17.0) | 18 (15.9) | 7 (17.1) | 2 (10.0) | 10 (22.7) |
|  | Other | 5 (2.4) | 4 (4.3) | 2 (2.5) | 0 | 0 | 3 (2.7) | 3 (7.3) | 0 | 1 (2.3) |
| Female | | 121 (59.0) | 43 (46.2) | 49 (62.0) | 14 (70.0) | 25 (53.2) | 64 (56.6) | 18 (43.9) | 14 (70.0) | 20 (45.5) |
| Education years | | 4.70 (4.1) | 4.60 (3.5) | 3.61 (2.9) | 5.80 (4.4) | 5.21 (4.3) | 5.29 (4.6) | 3.85 (2.4) | 5.80 (4.4) | 4.98 (4.2) |
| Neuropsychiatric symptoms (n, %) | | | | | | | | | | |
| Delusions | | 17 (8.3) | 11 (11.8) | 4 (5.1) | 3 (15.0) | 5 (10.6) | 11 (9.7) | 3 (7.3) | 3 (15.0) | 6 (13.6) |
| Hallucinations | | 17 (8.3) | 18 (19.4) | 3 (3.9) | 6 (30.0) | 5 (10.6) | 11 (9.7) | 7 (17.1) | 6 (30.0) | 8 (18.2) |
| Agitation | | 20 (9.8) | 9 (9.7) | 8 (10.3) | 2 (10.0) | 2 (4.3) | 11 (9.7) | 4 (9.8) | 2 (10.0) | 5 (11.4) |
| Depression | | 55 (27.0) | 33 (35.5) | 19 (24.4) | 8 (40.0) | 11 (23.4) | 29 (25.7) | 14 (34.2) | 8 (40.0) | 14 (31.8) |
| Anxiety | | 52 (25.5) | 26 (28.0) | 24 (30.8) | 6 (30.0) | 6 (12.8) | 21 (18.6) | 11 (26.8) | 6 (30.0) | 11 (25.0) |
| Elation | | 7 (3.4) | 3 (3.2) | 2 (2.6) | 2 (10.0) | 0 | 4 (3.5) | 0 | 2 (10.0) | 3 (6.8) |
| Apathy | | 21 (10.3) | 11 (11.8) | 5 (6.4) | 2 (10.0) | 6 (12.8) | 14 (12.4) | 4 (9.8) | 2 (10.0) | 6 (13.6) |
| Disinhibition | | 9 (4.4) | 1 (1.1) | 2 (2.6) | 1 (5.0) | 1 (2.1) | 7 (6.2) | 0 | 1 (5.0) | 1 (2.3) |
| Irritability | | 29 (14.2) | 6 (6.5) | 13 (16.7) | 1 (5.0) | 7 (14.9) | 15 (13.3) | 2 (4.9) | 1 (5.0) | 4 (9.1) |
| Motor symptoms | | 5 (2.5) | 2 (2.2) | 1 (1.3) | 0 | 1 (2.1) | 4 (3.5) | 0 | 0 | 2 (4.6) |
| NPI-10 number (mean, SD) | | 1.09 (1.6) | 1.29 (1.5) | 1.03 (1.6) | 1.55 (2.0) | 0.93 (1.5) | 1.07 (1.7) | 1.09 (1.2) | 1.55 (2.0) | 1.36 (1.7) |
| NPI-10 total score (mean, SD) | | 5.65 (12.1) | 6.86 (10.4) | 4.93 (10.9) | 8.9 (13.1) | 5.73 (10.6) | 5.90 (13.1) | 4.49 (6.1) | 8.9 (13.1) | 8.64 (13.2) |

Abbreviations: ADNC, Alzheimer’s disease neuropathological change; LBD, Lewy body disease in neocortical or limbic regions; LATE-NC, Limbic-predominant age-related TDP-43 encephalopathy neuropathologic change; CVD, cerebrovascular disease; CAA cerebral amyloid angiopathy. nLBD, neocortical-predominant LBD; lLBD, limbic-predominant LBD; NPI-10, neuropsychiatric inventory excluding appetite and sleep items.
